# Relationship between the use and types of helmets with facial injuries - a prospective study

**DOI:** 10.1590/0100-6991e-20223387-en

**Published:** 2022-12-09

**Authors:** CAROLINA CHAVES GAMA AIRES, HEITOR TAVARES DE ARAÚJO, ROSA RAYANNE LINS DE SOUZA, AÍDA JULIANE FERREIRA DOS SANTOS, RICARDO JOSÉ DE HOLANDA VASCONCELLOS, BELMIRO CAVALCANTI DO EGITO VASCONCELOS

**Affiliations:** 1 - Universidade de Pernambuco, Departamento de Cirurgia e Traumatologia Buco-Maxilo-Facial - Recife - PE - Brasil

**Keywords:** Facial Injuries, Head Protective Devices, Motorcycles, Accidents, Traffic, Traumatismos Faciais, Dispositivos de Proteção da Cabeça, Motocicletas, Acidentes de Trânsito

## Abstract

**Introduction:**

many studies have demonstrated the benefits of helmet to prevent and reduce severity of injuries in motorcyclists.

**Objective:**

the aim of the present study was to evaluate a possible relationship between the use of different types of helmets and the occurrence of facial injuries among victims of motorcycle accidents, seen at Hospital da Restauração, Recife/PE, Brazil.

**Materials and methods:**

demographic and trauma data were collected from hospitalized motorcycle accident victims with facial injuries from December 2020 to July 2021. Pearsons chi-square test was used to assess association between two categorical variables using a margin of error of 5%.

**Results:**

among the participants, the average age was 33.46 years. The age group between 18 and 29 years was the most prevalent. Most participants were male. 60.0% of motorcyclists used helmets at the time of the accident and of this percentage 37.6% used fixed full-face helmet, 16.5% open-face helmet and the other 5.9% articulated full-face helmet. 62.7% of participants had facial fractures. Among the fractures, those of the zygomatic-orbital complex were the most common fracture and were significantly associated with the use of helmets, especially with open-face helmet.

**Conclusions:**

the use of helmets was associated with a lower number of facial fractures among patients who were victims of motorcycle accidents. Fracture of the zygomatic-orbital complex was related to the absence of a helmet at the time of the accident, as well as the use of open-face helmets.

## RESUMO

**Introdução:**

muitos estudos têm demonstrado os benefícios do uso de capacete para prevenir e reduzir a gravidade das lesões em motociclistas.

**Objetivo:**

o objetivo do presente trabalho consistiu em avaliar uma possível relação entre o uso dos diversos tipos de capacete e a ocorrência do trauma facial entre vítimas de acidentes motociclísticos atendidos no Hospital da Restauração, Recife/PE, Brasil.

**Materiais e Métodos:**

os dados demográficos e referentes ao trauma foram coletados de vítimas de acidentes motociclísticos hospitalizados com lesões faciais durante o período de Dezembro de 2020 a Julho de 2021. O teste do qui-quadrado de Pearson foi utilizado para avaliar a relação entre duas variáveis categóricas utilizando uma margem de erro de 5%.

**Resultados:**

a idade média dos participantes foi 33,46 anos. A faixa etária entre 18 a 29 anos foi a mais prevalente. A maioria dos participantes eram do gênero masculino. 60,0% dos motociclistas utilizavam capacetes no momento do acidente e deste percentual 37,6% utilizavam capacete fechado, 16,5% capacete aberto e os outros 5,9% capacete escamoteável. 62,7% dos participantes tiveram fraturas faciais. Dentre as fraturas, as do complexo zigomático-orbitário foram as mais prevalentes e foram associadas com o uso de capacetes, especialmente os abertos.

**Conclusões:**

o uso de capacetes foi associado com um menor número de fraturas faciais entre os paciente vítimas de acidentes motociclísticos. A fratura do complexo zigomático-orbitário foi relacionada com a ausência de capacete no momento do acidente, bem como com o uso de capacetes abertos.

## INTRODUCTION

Motorcyclists are often involved in fatal accidents and serious injuries, the rate of injuries per traveled kilometer being higher than for any other type of vehicle^1^. Motorcycle accidents are responsible for 23% of global road traffic deaths and more than half of deaths in countries where motorcycles are the dominant means of transport^2^. These high rates can be explained by the inherent instability of the vehicle and the low level of protection offered when compared to automobiles^3^. The inherent vulnerability of this type of vehicle makes motorcyclists susceptible to high-impact collisions^4^.

In Brazil, the number of motorcyclists involved in traffic accidents is gradually surpassing that of other road users, and motorcyclists are more vulnerable to injury than occupants of other motor vehicles. In addition, motorcycles are increasingly used to transport passengers (“mototaxis”) and for commercial purposes (“motoboys”). Resolution 203 of the 2006 Brazilian Traffic Code (CTB) made it mandatory for motorcyclists to wear helmets on public roads^5^.

Facial injuries, including fractures, have serious implications for the quality of life of victims of traffic accidents. The institution of laws that require the use of seat belts in cars and helmets in motorcycles directly impacts the incidence of these injuries. The physical, emotional, and functional consequences of these accidents can result in permanent deformities^5^.

Many studies have consistently demonstrated the benefits of helmet use to prevent head lesions and reduce mortality and injury severity in motorcyclists^3^. The use of this safety equipment can reduce the risk of head injury by up to 69%, and death, by 42%^2^. However, both in developed and developing countries, there is still resistance to laws regarding the mandatory use of helmets, and the debate on the effectiveness of helmet use in reducing the occurrence and severity of facial trauma is still not well documented^3^.

Various types of helmets are available for motorcyclists. According to the CTB, three types of helmets can be used on Brazilian roads: open helmets, fully closed helmets, and retractable or articulated closed helmets. Facial injuries seem to be more frequent and severe in riders who wear open helmets, as this type of helmet leaves some parts of the face unprotected. However, the evidence on face protection provided by the different types of available headgear is still inconsistent^3^ and requires further research that may or may not support this hypothesis. In this context, the objective of this study was to evaluate a possible relationship between the use of different types of helmets and the occurrence of facial trauma.

## MATERIALS AND METHODS

This was a cross-sectional, prospective, observational study carried out in a public hospital in the city of Recife, state of Pernambuco, Brazil. Patients from the public health network treated by the Oral and Maxillofacial Surgery team at Hospital da Restauração (HR) were selected from December 2020 to July 2021. This study was conducted according to the guidelines of the Strengthening the Reporting of Observational Studies in Epidemiology (STROBE) for observational studies^6^. 

We included victims of motorcycle accidents admitted to HR who had some type of facial injury. Patients responded to a questionnaire regarding trauma history, demographics, and the use of different types of helmets. The medical records of these patients were also analyzed to obtain information on the presence of extra and intraoral lesions, as well as questions regarding diagnosis, based on clinical and tomographic examinations. Children and adolescents under 18 years of age were not included in the research. Patients who were unable to answer the questionnaire due to their clinical condition, or who did not know how to answer the questions, and those who had incomplete medical records were excluded from the sample. 

Statistical analysis of data was performed using the Statistical Package for the Social Sciences (SPSS) software, v.25.0. The sample size was calculated based on the number of patients seen at HR in 2018 (1,982,887 patients), obtained from data from the Health Department of the state of Pernambuco. Using the parameters of 95% confidence interval and 5% margin of error, the calculated ideal sample size was 246 patients. Data were descriptively analyzed using absolute frequencies and percentages for the following categorical variables: mean, median, and standard deviation. To assess the association between two categorical variables, we used the Pearson’s chi square test when the condition for using the chi square test was not met. The margin of error used in the decision of the statistical tests was 5%. 

The Ethics in Research Committee of the University of Pernambuco approved the study under number 4,688,284. All patients were informed about the objectives of the study and signed an informed consent form. The procedures of this study were conducted according to the Declaration of Helsinki.

## RESULTS

The age of the patients studied ranged from 18 to 76 years, with a mean of 33.46 years, standard deviation of 11.95 years, and median of 31.00 years. Table 1 shows the characteristics of the sample. The majority (88.6%) were male, and the age group 18 to 29 years was the most prevalent, accounting for 47.5%, followed by the group 30 to 39 years (25.1%), the percentages of the other two age groups ranging from 12.9% to 14.5%. The two regions of the state with the highest frequencies of participants corresponded to the Agreste, with 43.5%, and the Recife Metropolitan Region (RMR), with 40.4%.

**Table 1:** Occurrence of fracture according to sample characteristics.

	Fracture			
Variable	Yes	No	Total	p-value^1^
	n (%)	n (%)	n (%)	
Sex				0.192
Male	145 (64.2)	81 (35.8)	226 (100.0)	
Feminine	15 (51.7)	14 (48.3)	29 (100.0)	
Age group				0.698
18-29	79 (65.3)	42 (34.7)	121 (100.0)	
30-39	38 (59.4)	26 (40.6)	64 (100.0)	
40-49	21 (56.8)	16 (43.2)	37 (100.0)	
≥50	22 (66.7)	11 (33.3)	33 (100.0)	
Origin				0.080
RMR	58 (56.3)	45 (43.7)	103 (100.0)	
Outside the RMR	102 (67.1)	50 (32.9)	152 (100.0)	
Occupation/ profession				0.141
Farmer	58 (74.4)	20 (25.6)	78 (100.0)	
Student	9 (64.3)	5 (35.7)	14 (100.0)	
Motoboy	17 (54.8)	14 (45.2)	31 (100.0)	
Mason	12 (60.0)	8 (40.0)	20 (100.0)	
Other	64 (57.1)	48 (42.9)	112 (100.0)	
Total Group	160 (62.7)	95 (37.3)	255 (100.0)	

^1^Pearson’s Chi-square test.

Regarding the occupations of those surveyed, the highest frequencies standing out were farmer (30.6%), motoboy (12.2%), and bricklayer (7.8%); 5.5% were students, a category separated not because of its greater frequency, but because of its peculiarity; the other occupations were grouped and totaled 43.9% of the sample.

Most motorcyclists (60.0%) wore a helmet at the time of the accident. Of those, 37.6% used a closed helmet, 16.5% an open one, and the other 5.9%, a retractable helmet. Alcohol consumption before the accident was present in 30.2% of the sample. Of the 102 participants who were not wearing a helmet at the time of the accident, only 15 were female. We observed a significant association (p<0.001, Pearson’s Chi-square test) between the use of helmet and the origin of the researched individual, showing that the percentage who used the helmet was higher among the residents of the RMR than among those who were not from the RMR (73.8% x 50.7%).

**Figura 1:**
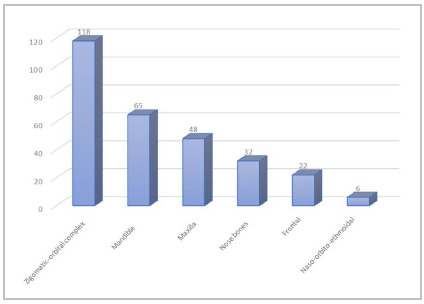
Prevalência das fraturas faciais.

Regarding the prevalence of fractures, the majority (62.7%) had fractures and some soft tissue injury and the remaining 37.3% had only soft tissue injuries; among the 160 who had a fracture, the most frequent types of fracture were 118 of the zygomatic-orbital complex (ZOC), 65 mandibular, 48 maxillary, and 32 fractures of the nose bones. Fractures of the frontal bone and of the naso orbito ethmoidal region occurred in smaller numbers, as can be seen in Figure 1.

Table 1 presents the occurrence of fractures according to the sample characteristics. We highlight the higher percentage of fractures among males than females (64.2% x 51.7%), as well as higher among those coming from locations outside the metropolitan region (RMR) than those coming from the RMR (67.1% vs. 56.2%). The fracture percentage was higher among farmers (74.4%) and lower among motoboys (54.8%). However, for the fixed margin of error (5%), we found no significant associations (p>0.05) between the occurrence of fracture and the sample characteristics.

Table 2 shows the significant association between the occurrence of fractures with the use and type of helmet, and for these variables, the higher percentage of fractures among those who did not use helmets (75.5%). Regarding the type of helmet, there were more fractures among those surveyed who used open helmets (78.6%), followed by those who used retractable ones (66.3%), and a lower percentage among those who used closed helmets (41.7%).

**Table 2:** Occurrence of fracture according to the use and type of helmet.

	Fracture			
Variable	Yes	No	Total	p-value^1^
	n (%)	n (%)	n (%)	
Helmet use				0.001*
Yes	83 (54.2)	70 (45.8)	153 (100.0)	
No	77 (75.5)	25 (24.5)	102 (100.0)	
Group Total	160 (62.7)	95 (37.3)	255 (100.0)	
Helmet type				<0.001*
Open - face	33 (78.6)	9 (21.4)	42 (100.0)	
Fixed full-face	40 (41.7)	56 (58.3)	96 (100.0)	
Articulated full-face	10 (66.7)	5 (33.3)	15 (100.0)	
Group Total	83 (54.2)	70 (45.8)	153 (100.0)	

*Significant association at 5%; ^1^Pearson’s Chi-square test.

ZOC fractures were more common in males than in females (75.9% vs. 53.3%). When comparing ZOC fractures with some characteristics of the sample, we found a significant association between its occurrence and the age groups. These fractures were more common among individuals aged between 40 and 49 years (90.5%) and above 50 (95.5%). There was a significant association of ZOC fractures and the use and types of helmets (p<0.05, Table 3): the percentage with fracture was higher among those who did not use a helmet than among those who used one (81.8% vs. 66.3%), and higher among those who used open helmets (81.8%).

**Table 3:** ZOC fracture according to the use and type of helmet.

	ZOC fracture			
Variable	Yes	No	Total	p-value^1^
	n (%)	n (%)	n (%)	
Helmet use				0.025*
Yes	55 (66.3)	28 (33.7)	83 (100.0)	
No	63 (81.8)	14 (18.2)	77 (100.0)	
Group Total	118 (73.8)	42 (26.3)	160 (100.0)	
Helmet type				0.047*
Open - face	27 (81.8)	6 (18.2)	33 (100.0)	
Fixed full-face	23 (57.5)	17 (42.5)	40 (100.0)	
Articulated full-face	5 (50.0)	5 (50.0)	10 (100.0)	
Group Total	55 (66.3)	28 (33.7)	83 (100.0)	

*Significant association at 5%. ^1^Pearson’s Chi-square test.

Sex displayed a significant association with the occurrence of mandibular fracture (p<0.05), with a significantly higher percentage observed among male patients (66.7%). As shown in Table 4, there was a significant association between helmet use and the occurrence of mandibular fracture, with the percentage of individuals with mandibular fracture being higher among those who wore helmets (50.6%). Differently from what happened with the ZOC fractures, there was no statistical difference between the occurrence of mandibular fractures and the types of helmets used.

**Table 4:** Mandibular fracture according to the use and type of helmet.

	Mandibular fracture			
Variable	Yes	No	Total	p-value^1^
	n (%)	n (%)	n (%)	
Helmet use				0.008*
Yes	42 (50.6)	41 (49.4)	83 (100.0)	
No	23 (29.9)	54 (70.1)	77 (100.0)	
Group Total	65 (40.6)	95 (59.4)	160 (100.0)	
Helmet type				0.835
Open - face	18 (54.5)	15 (45.5)	33 (100.0)	
Fixed full-face	19 (47.5)	21 (52.5)	40 (100.0)	
Articulated full-face	5 (50.0)	5 (50.0)	10 (100.0)	
Group Total	42 (50.6)	41 (49.4)	83 (100.0)	

*Significant association at 5%. ^1^Pearson’s Chi-square test.

There were no significant association (p>0.05) between the occurrence of maxillary fracture and the other variables related to sample characteristics. As shown in Table 5, we also observed no significant associations (p>0.05) between the occurrence of maxillary fracture, neither with the use nor with the type of helmet used.

**Table 5:** Maxillary fractures according to the use and type of helmet.

	Maxillary fractures			
Variable	Yes	No	Total	p-value^1^
	n (%)	n (%)	n (%)	
Helmet use				0.317
Yes	22 (26.5)	61 (73.5)	83 (100.0)	
No	26 (33.8)	51 (66.2)	77 (100.0)	
Group Total	48 (30.0)	112 (70.0)	160 (100.0)	
Helmet type				0.586
Open - face	8 (24.2)	25 (75.8)	33 (100.0)	
Fixed full-face	10 (25.0)	30 (75.0)	40 (100.0)	
Articulated full-face	4 (40.0)	6 (60.0)	10 (100.0)	
Group Total	22 (26.5)	61 (73.5)	83 (100.0)	

^1^Pearson’s Chi-square test.

## DISCUSSION

The use of different types of helmets influences the occurrence of different facial traumas. In general, the high rates of injuries caused by motorcycle accidents can be explained by the inherent instability of the vehicle and the low level of protection offered when compared with automobiles. In addition, the non-use of personal protective equipment and possible consumption of alcohol influence the individual’s piloting ability^3^.

Most of the motorcyclists evaluated in this study were young men, mainly between the ages of 18 and 29 years, and living in areas outside the metropolitan region. This fact corroborates several studies found in the literature, both in terms of sex^7^ ^-^ ^9^ and age^10^ ^,^ ^11^. A systematic review on risk factors involving traffic accidents encompassing 2,703 studies and including a total of 422,244 patients identified sex, age, and origin as risk factors for the occurrence of facial injuries resulting from traffic accidents (p<0.05)^12^. Despite the accessibility, maneuverability, and cost-effectiveness of motorcycles making them a popular choice of transportation in busy urban centers, the use of this vehicle requires a high degree of coordination, proper judgment, and experience for safe driving^4^. These factors may explain the more frequent involvement of young individuals in this type of accident.

The fact that most of the participants came from rural areas corroborates numerous epidemiological studies on the use of motorcycles^12^. Low fuel consumption, easy access to these vehicles, and ease of transit between cities make the use of motorcycles quite common, especially in rural areas. Unfortunately, this is not accompanied by proper monitoring and enforcement of motorcyclists. Therefore, the large number of motorcycle accidents tend to be common in these regions^13^. Although the state of Pernambuco is not one of the most motorized, mortality rates resulting from traffic accidents are proportionately high when compared with other Brazilian states, largely due to motorcycle accidents^10^.

Head trauma and facial injuries are critical factors in the morbidity and mortality patterns of motorcycle accidents, and the protective effect of helmets on victims of these accidents is already well established^14^. Although most studies find strong evidence that helmets protect against injuries during traffic collisions, some have observed a positive association between helmet use and neck injuries^15^. However, data from a systematic review did not support this hypothesis^16^.

The results of the present study evidenced the importance of the helmet as an individual protection equipment (IPE), since the rate of facial fractures was significantly higher among motorcyclists who were not helmeted, corroborating previous studies^14^ ^,^ ^15^ ^,^ ^17^. A recent meta-analysis carried out from 22 studies also showed a greater number of fractures among motorcyclists without helmets, with an adjusted hazard ratio of 0.32 (95% CI 0.17 - 0.62)^3^. Thus, the importance of using this protective equipment is evident, as well as the need to inspect and guarantee its compulsory use.

In Brazil, resolution 453/2013 of the Brazilian Traffic Code (CTB) made the use of helmets mandatory for motorcyclists and passengers on public roads. Helmets must be certified by the National Institute of Metrology, Quality and Technology (INMETRO), responsible for ensuring the safety of the equipment. Only three types of helmets are approved in Brazil: full-face, open-face, and retractable or articulated, helmets. The use of goggles is also mandatory when wearing open-face helmets^5^. The CTB recommends the use of other IPE, such as appropriate clothing and footwear, clothing with reflective strips, markers, and side handles, and line trimmers, also known as ‘Antennas for cutting kites` strings’^18^. Despite the obligation provided by law, 40% of the research participants who suffered motorcycle accidents were not wearing a helmet at the time of trauma. This implies that inspection is still a crucial point in preventing injuries.

In addition to the benefit of individual protection, the use of helmets also benefits society. A reduction in mortality rates, lower Injury Severity Score (ISS) scores, lower ICU admission rates, fewer traumatic brain injuries, shorter hospital stays, fewer surgical procedures, especially those resulting from facial injuries, and lower hospitalization costs is expected when motorcyclists make the correct use of this IPE^14^ ^,^ ^17^. A previous study carried out at HR found that of the US$ 51,285.00 spent on the use of osteosynthesis materials to treat facial fractures, US$ 37,246.89 was spent on victims of motorcycle accidents^10^. Such facts represent the impact of these injuries in terms of public health.

Regarding facial trauma, the results obtained in the study corroborate previous studies that related high-impact trauma resulting from motorcycle accidents with high rates of lacerations, soft tissue injuries, and facial fractures^4^ ^,^ ^13^. All patients evaluated had some soft tissue injury, and 62.7% of all participants sustained facial fractures. This result is similar to that obtained by Oginni et al. (2006)^4^, who studied 367 cases of motorcycle accidents and observed 221 patients (60%) with 338 facial fractures, a proportion of 1.5 fractures per patient.

Among facial fractures, the ones of the zygomatic-orbital complex were the most prevalent in the present study, followed by mandibular ones. Some authors suggest that the more projected position of the zygoma makes it more vulnerable during collisions^13^ ^,^ ^10^ ^,^ ^19^. Thus, the importance of the helmet is evident, since it was possible to associate the use of this IPE with a lower occurrence of fractures of the zygomatic-orbital complex. In addition, we also observed that the use of open helmets was statistically associated with this fracture pattern.

A recent meta-analysis evaluated the occurrence of fractures in the three facial thirds with the use of helmets and found that patients without helmets had a significantly higher number of fractures of the middle and upper third of the face, in addition to lower trauma severity scores. In this meta-analysis, fractures in the upper and middle thirds of the face were significantly more common, with an adjusted hazard ratio of 0.43 (95% CI 0.24 - 0.78) for the upper third and 0.70 (95% CI 0.50 - 0.97) for the middle third. On the other hand, no significant difference was observed between the groups with and without helmets when analyzing the lower third of the face^3^. In other words, this meta-analysis was unable to state that the use of a helmet was effective in reducing the occurrence of lower facial fractures. In the present study, we found that riders with helmets suffered more mandibular fractures than individuals without helmets.

As for the types of helmets used and facial fractures, the only statistically significant association found was between fractures of the zygomatic complex and the use of open helmets. Some authors suggest that since helmets were made to protect the skull, this would explain greater protection of the upper and middle thirds of the face^19^. On the other hand, open helmets in theory leave the lower third of the face more vulnerable, which could lead to higher rates of mandibular and even maxillary fractures. We did not find such association, though. According to Cavalcante et al.^3^, despite the well-established relationship between the use of helmets and a lower number of facial fractures and trauma severity, the current literature is still not clear whether the type of helmet, open or whole, interferes with the occurrence and severity of facial injuries. 

One of the limitations of this study is the inability to infer a cause-and-effect relationship between the absence of a helmet and the occurrence of facial traumas and fractures, given its methodological design. However, the present work brings relevant information on the subject and emphasizes the vulnerability of motorcyclists, mainly due to the absence of IPE. Studies show that the risk of being punished reduces the probability of being involved in accidents and impacts behavioral changes in drivers who engage in risky behaviors^20^. Greater inspection and enforcement of traffic laws are necessary to reduce morbidity and mortality rates associated with motorcycle accidents^13^.

## CONCLUSION

Helmet use was associated with a lower number of facial fractures among motorcycle accident victims. Among the most prevalent fractures, the zygomatic-orbital complex one was related to the absence of a helmet at the time of the accident, as well as with the use of open helmets. There was no association between the other types of facial fractures and the use of open-face and/or articulated full-face helmets. The use of helmets influences the occurrence of facial trauma and their compulsory use should be more monitored, in an attempt to reduce these numbers.
